# Nutritional management of neonates who undergo major surgery for gastrointestinal disorders: a joint position paper of the Italian Society of Neonatology (SIN), the Italian Society of Pediatric Surgery (SICP), and the Italian Society of Pediatric Nutrition (SINUPE)

**DOI:** 10.1186/s13052-025-02188-0

**Published:** 2026-01-10

**Authors:** Domenico Umberto De Rose, Arianna Aceti, Nadia Liotto, Laura Morlacchi, Elena Maggiora, Simonetta Costa, Andrea Conforti, Maria Lorella Giannì, Maria Elisabetta Baldassarre, Giovanna Verlato, Pasqua Betta, Nunzia Decembrino, Alessandra Consales, Carla Ottaviano, Maria Grazia Scuderi, Lorenza Putignani, Giorgio Fava, Luigi Orfeo, Massimo Agosti, Guglielmo Salvatori, Irma Capolupo, Collaborators of the Working Group on Nutrition of Surgical Newborn, Cecilia Capetti, Martina Capitanio, Martina Ruscelli, Federica Natalizio, Stefano Pellicani, Andrea Dotta, Luca Maggio

**Affiliations:** 1 Neonatal Intensive Care Unit, “Bambino Gesù” Children’s Hospital IRCCS, Rome, Italy; 2 Department of Medical and Surgical Sciences, University of Bologna, Bologna, Italy; 3 Neonatal Intensive Care Unit, IRCCS AOUBO, Bologna, Italy; 4 Neonatal Intensive Care Unit, Fondazione IRCCS Ca’ Granda Ospedale Maggiore Policlinico, Milan, Italy; 5 Division of Neonatology, Woman and Child Department, “F. Del Ponte” Hospital, ASST-Settelaghi, Varese, Italy; 6 Neonatology Unit of the University, Sant’Anna Hospital, Città della Salute e della Scienza, Turin, Italy; 7 Department of Public Health and Pediatric Sciences, University of Turin, Turin, Italy; 8 Neonatology and Neonatal Intensive Care Unit, Policlinico Casilino, Rome, Italy; 9 Neonatal and Pediatric Surgery Unit, “Bambino Gesù” Children’s Hospital IRCCS, Rome, Italy; 10 Department of Interdisciplinary Medicine, Neonatology and NICU Section, University “Aldo Moro”, Bari, Italy; 11 Neonatal Intensive Care Unit, University Hospital of Padua, Padua, Italy; 12 Azienda Ospedaliera-Universitaria Policlinico Vittorio Emanuele, Presidio Ospedaliero Gaspare Rodolico, Catania, Italy; 13 Department of Clinical Sciences and Community Health, University of Milan, Milan, Italy; 14 Neonatal Intensive Care Unit, “San Camillo Forlanini” Hospital, Rome, Italy; 15 Microbiomics Unit, “Bambino Gesù” Children’s Hospital IRCCS, Rome, Italy; 16 Pediatric Surgery Unit, Fondazione IRCCS Ca’ Granda Ospedale Maggiore Policlinico, Milan, Italy; 17 Neonatal Intensive Care Unit, “Gemelli Isola – Isola Tiberina” Hospital, Rome, Italy; 18 Department of Medicine and Surgery, University of Insubria, Varese, Italy

## Abstract

Neonates undergoing major gastrointestinal surgery for congenital or acquired disorders require complex nutritional management to support their growth and recovery. Prolonged fasting can be detrimental, necessitating timely and appropriate nutritional support. This joint position paper by the Italian Society of Neonatology, the Italian Society of Pediatric Surgery, and the Italian Society of Pediatric Nutrition aims to provide evidence-based suggestions for the nutritional care of these vulnerable infants, addressing the lack of robust randomized controlled trials in this field through expert opinion. A panel of experts in neonatology, paediatric surgery, and paediatric nutrition across Italy reviewed the literature by searching the PubMed database (1990- September 2024) using specific keywords. English-language papers were analysed without restrictions on study design or outcomes. Identified references were cross-checked, and additional relevant literature was included based on expert knowledge. The panel formulated suggestions based on the available evidence and clinical expertise. The position paper provides specific suggestions for various aspects of nutritional management, including the timing and modalities of enteral nutrition (EN), the choice of milk (prioritizing human milk), vitamin and trace element supplementation, and condition-specific guidance for gastrointestinal disorders such as oesophageal atresia, congenital diaphragmatic hernia, chylothorax, intestinal atresia, abdominal wall defects, Hirschsprung disease, necrotizing enterocolitis, and intestinal failure. Early EN (within 48 hours post-surgery) is generally advised and then tailored according to feeding tolerance. The paper also emphasizes the importance of monitoring micronutrient deficiencies and promoting oral feeding skills. This joint position paper offers a comprehensive and multidisciplinary approach to the nutritional management of neonates undergoing major gastrointestinal surgery. Recognizing the limitations of current evidence, these suggestions aim to standardise and optimise nutritional care, based on available data and expert consensus, ultimately improving outcomes for this high-risk population. The paper highlights the need for individualised nutritional strategies, careful monitoring, and further research in this challenging area of neonatal care.

## Introduction

The term ‘‘surgical newborn” refers to a neonate who undergoes surgery during the first days of life, due to a variety of diagnoses: congenital gastrointestinal disorders that include abdominal wall defects, intestinal atresias, Hirschsprung disease and other colorectal malformations; complex disorders such as oesophageal atresia (OA) and congenital diaphragmatic hernia (CDH); acquired conditions such as necrotizing enterocolitis (NEC) and other conditions that can lead to bowel resection and short bowel syndrome (SBS). The nutritional support that these newborns need is part of a complex path of care, including pre-operative (stabilization and multidisciplinary management), peri-operative (analgesia, correct fluid management, maintenance of normothermia), and post-operative interventions (weaning from analgesia, enterostomy care) [1].

**Fig. 1:**
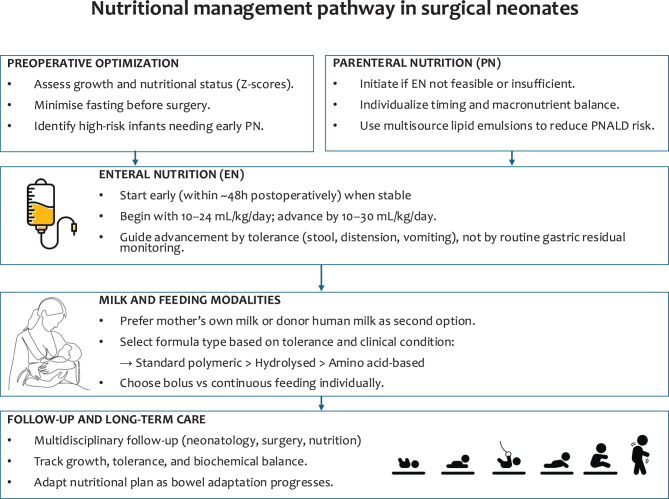
Flowchart summarizing the main steps of perioperative nutritional management in surgical neonates, integrating parenteral and enteral strategies within an expert-based framework necessitated by the rarity of these conditions

The relatively low incidence of each of these conditions limits the possibility of having clear evidence about the correct nutritional management of these children due to the lack of randomized controlled trials in this field. Therefore, we aimed to collect available data from the literature and provide position statements shared by a writing consensus group, including experts in neonatology, paediatric surgery, and paediatric nutrition. A summary of main recommendations is provided in Fig. 1.

## Methods

The development of this position paper involved the participation and contribution of members of three scientific societies: neonatologists from the board of the Study Group of Surgical Newborn and the Study Group of Neonatal Nutrition and Gastroenterology of the Italian Society of Neonatology (SIN), paediatric surgeons from the Study Group of the Surgical Newborn of the Italian Society of Paediatric Surgery (SICP), and neonatologists from the Italian Society of Paediatric Nutrition (SINUPE). The panel of experts was chosen based on their expertise and relevant clinical practice, representing the main institutions that care for surgical newborns.

To evaluate available literature on this topic, the PubMed database was searched by combining terms related to nutrition, surgery, and each gastrointestinal surgical condition in newborns. All English-language papers that were recovered and released from 1990 to 30 September 2024 were analysed without imposing restrictions on date, country, study design, outcomes, or inclusion/exclusion criteria. The reference lists of the identified papers were further checked, and each author identified additional references for this review based on their expertise in the relevant topic.

## Parenteral Nutrition

A fasting period is necessary, at least in the perioperative period, but newborns grow very quickly and have a body composition different from that of children and adults [2], so they cannot tolerate prolonged periods of fasting. Therefore, parenteral nutrition (PN) may serve as their sole nutrient source [3].

PN is essential for supporting growth, recovery, and development, while preventing malnutrition; in most cases, it is a temporary intervention to bridge infants to full enteral feeding (FEF). However, in severe cases (e.g., in infants with short bowel syndrome), long-term PN may be required, and surgical infants are especially vulnerable to nutritional and metabolic complications, as well as impaired growth, both in the acute/post-acute phase and over the long term. In such cases, the infant is at increased risk for various complications, including infections (due to indwelling central venous access), nutrient imbalances (both deficiencies and excesses of specific nutrients), metabolic disturbances (such as glycemia/electrolyte imbalances, fat overload, or metabolic acidosis), and liver complications [4, 5]. Prolonged PN may lead to liver dysfunction, a condition known as parenteral nutrition-associated liver disease (PNALD), particularly due to lipid overload [4].

In the PEPaNIC trial, which is the largest randomized controlled trial (RCT) on nutrition in critically ill children (*n* = 1440), the authors showed potential harm by initiating early PN (within 24 hours) to supplement insufficient or contraindicated enteral nutrition (EN), as compared to withholding PN until one week after admission in intensive care unit (late PN). Although PEPaNIC did not include preterm infants, term infants (including neonates) were included and about 8% of included patients underwent abdominal surgery [6].

A secondary analysis of the PEPaNIC trial compared early PN versus late PN, focusing specifically on critically ill term neonates: withholding PN during the first week of critical illness in term neonates may improve short-term outcomes such as infection rates and intensive care stay, but at the cost of increased hypoglycemia [7]. These findings do not contradict the essential role of PN in surgical neonates but rather emphasize that PN should be initiated only when EN is not feasible or sufficient. While PN remains a cornerstone of nutritional support to ensure growth and recovery, the optimal timing of its initiation should be individualised based on the infant’s clinical condition, the feasibility of EN, and metabolic tolerance. The PEPaNIC trial primarily suggests that unnecessary early initiation of PN may be avoided in some situations, but does not question its essential role when nutritional needs cannot be met otherwise.

Concerning macronutrients, carbohydrates and lipids are the primary energy sources, while proteins are critical for tissue synthesis. Maintaining an appropriate ratio between energy substrates and protein intake is vital to promote an anabolic utilization of amino acids and to minimize their catabolism [8].

Major surgical procedures in newborns typically result in a moderate and immediate increase in energy expenditure, which returns rapidly to baseline levels. No further elevation in energy expenditure is observed during the first 5 to 7 postoperative days. Consequently, monitoring caloric intake during the postoperative period is essential to ensure appropriate nutritional support [9].

There is no clear evidence of the need to provide a greater caloric intake to newborns undergoing surgery, given the conflicting results in the literature in relation to the type of operation and the small samples analysed [10]. Although energy requirements appear to increase by 15 to 25% in the first 4 hours after major abdominal surgery and then return to normal values within 24 postoperative hours, after the first 24 postoperative hours no changes in energy requirements have been observed [11]. At the moment, we know that growth is hindered during acute stress, so the choice of caloric intake must be personalized, referring to the weight trend and hemodynamic state of the newborn.

To mitigate the risk of hyperglycemia in the immediate postoperative period, a temporary reduction in carbohydrate administration may be considered (5.8–11.5 g/kg/day for preterm newborn and 3.6–7.2 g/kg/day for term newborn), with close monitoring of glycaemic response and gradual adjustments as needed to avoid insulin therapy. Carbohydrate intake should not exceed the values recommended by current neonatal PN guidelines (17.3 g/kg/day) [12].

Lipid emulsions are a key component in PN for surgical infants: they provide concentrated source of calories (contributing to approximately 30–50% of the total energy intake), essential fatty acids (necessary for cell membrane structure and immune function) and facilitate the absorption of fat-soluble vitamins, critical for growth, immune function, and bone health. For many years, soybean-based lipid emulsion has been the principal source of intravenous fat. Traditional emulsions, rich in omega-6 (ω-6) polyunsaturated fatty acids (PUFAs) and poor in omega-3 (ω-3) PUFAs, have been replaced by newer, more balanced formulations using multiple lipid sources: soybean oil (which is a source of essential fatty acids), medium-chain triglycerides (which are rapidly metabolized lipids), olive oil (which is rich in monounsaturated fats and naturally contains the antioxidant vitamin E) and fish oil (which is rich of ω-3 PUFAs, known for their anti-inflammatory properties and potential to reduce the risk of PNALD), which are known for their anti-inflammatory effect [13, 14].

Concerning protein intake, although no specific guidelines exist for parenteral protein requirements in surgical neonates, increased protein provision may be indicated in cases with additional losses, such as those associated with enterostomies or surgical drains [15, 16].

Vitamins, minerals, and trace elements are essential for metabolic functions, immune competence, and overall growth and development. In newborns undergoing surgery, timely supplementation of these micronutrients is critical, particularly in infants with enterostomies or those who have undergone intestinal resection, as they are especially susceptible to deficiencies, most notably zinc, due to increased losses and elevated physiological demands. In this high-risk population, ensuring adequate zinc supplementation is of critical importance to support postoperative recovery and promote optimal growth [17, 18].

### Take home messages

1. *The panel highlights that PN is vital for surgical infants to ensure growth and recovery, but it carries risks of serious complications such as infections, metabolic disturbances, and PNALD due to prolonged use.*
2. *Properly balancing carbohydrates, lipids, and proteins is critical in managing PN, especially in the postoperative phase, where the energy expenditure does not increase significantly and hyperglycemia may occur.*
3. *New multicomponent lipid emulsions combining various oil sources, including medium-chain triglycerides and fish oil, improve nutrient delivery and may mitigate the risk of liver complications associated with traditional soybean-based emulsions.*

## Enteral Nutrition: timing, assessment of feeding tolerance and modalities

Traditionally, fasting after abdominal surgery (until gastric residuals – GR – became non-biliary or paralytic ileus resolved) was considered essential to prevent feeding intolerance and complications like anastomotic leaks [19, 20].

However, prolonged fasting ( > 4 days) has been linked to increased intestinal permeability, raising the risk of intestinal injury, bacterial translocation, and antigen permeability upon later refeeding, which could contribute to systemic inflammation [21].

Total PN induces villous atrophy [22], whereas EN supports mucosal integrity, motility, and microbiota through hormonal and trophic effects [23–26].

Two recent meta-analyses in neonates and children found that starting EN within 48 hours after surgery reduced surgical site infections and shortened time to first stool and hospital stay without increasing the risk of anastomotic leaks, wound dehiscence, or feeding intolerance [27, 28].

Consequently, the Enhanced Recovery after Surgery (ERAS) Society recommends initiating EN within 48 hours postoperatively, regardless of surgical type, GR, or bowel movements. However, the guidelines do not specify when to increase initial feeding volumes or the best method for progression [1].

Based on available trials and expert opinions, our proposal is to start EN at 10–24 mL/kg/day within 48 hours postoperatively, with subsequent increases ranging from 10 to 30 mL/kg/day [20, 29–31].

Feeding progression should follow tolerance indicators like stool output and bowel movements. The tolerated output volume ranges from 20 to 50 mL/kg/day, and exceeding these levels may necessitate reducing or temporarily halting feedings. A 6–10/day bowel movement frequency is also considered a key tolerance indicator [30–32].

Therefore, while also ERAS guidelines endorse early initiation of EN, they do not provide explicit recommendations regarding starting volumes or rates of advancement. The thresholds proposed in this manuscript for feeding volumes and stool output are intended as pragmatic, expert-based guidance rather than evidence-based standards, acknowledging that no universal definition of feeding tolerance currently exists. These values should therefore be interpreted as flexible clinical suggestions to support individualised decision-making rather than prescriptive cut-offs.

Additional indicators of feeding intolerance include abdominal distension, vomiting, blood in the stool, and GR, but no standardised criteria exist for these symptoms. Recommendations of the European Society of Pediatric Gastroenterology, Hepatology and Nutrition (ESPGHAN) and a recent meta-analysis indicate that routine monitoring of GR is not useful and may delay nutritional recovery. Therefore, GR assessments should not be part of routine practice but should be conducted only if other signs of intolerance exist [33].

Oral nutrition should be encouraged as soon as possible to stimulate swallowing reflexes and prevent oral aversion. However, when suckling is not feasible, nasogastric or orogastric tubes are used for EN, with two main feeding strategies. Intermittent EN, which simulates a normal feeding pattern by delivering feedings in short intervals, is considered more physiological, as it stimulates intestinal hormone secretion and promotes bile acid release. Continuous EN provides a constant infusion over 24 hours, which increases the contact time between nutrients and the intestinal epithelium, enhancing digestion and absorption while reducing osmotic load [34–37]. The choice between continuous and bolus feeding should be individualised, taking into account each infant’s clinical stability, intestinal motility, and tolerance. In some cases, a combination approach (for example, continuous feeding during the night and intermittent feeding during the day) may optimize tolerance and growth.

No conclusive evidence supports one modality over the other in surgically treated neonates. A notable issue with continuous EN is the loss of calories, particularly lipids, through the feeding system, resulting in an estimated reduction of about 28 Kcal/kg/day [38, 39]. In the absence of clear advantages, intermittent EN is generally preferred, with continuous EN used as an adjunct during nighttime or in cases of accelerated intestinal transit, such as in neonates with short bowel syndrome.

Finally, post-pyloric feeding can be considered for critically ill children at high risk of aspiration or requiring frequent fasting for surgery or procedures, as suggested by recommendations from European Society of Pediatric and Neonatal Intensive Care (ESPNIC) [40].

### Take home messages

1. *The panel suggests initiating early EN within 48 hours postoperatively, with feeding progression guided by tolerance indicators such as stomal output and bowel movement frequency. This approach aims to avoid the detrimental effects of prolonged fasting and promote gut health.*
2. *The panel recommends starting EN at 10–24 mL/kg/day, with daily increases of 10–30 mL/kg/day, according to individual tolerance and clinical stability, to ensure safe and gradual feeding advancement.*
3. *Routine monitoring of GR as a primary indicator of feeding tolerance should be avoided. Instead, tolerance should be assessed based on clinical signs like abdominal distension, vomiting, and blood in the stool, reserving GR assessment for cases with other intolerance signs. This recommendation aims to prevent unnecessary delays in nutritional advancement.*
4. *The choice of feeding modality (continuous vs bolus, or a combination of both) should be individualised according to the infant’s clinical condition and tolerance, as no single approach has proven to be superior to others. When feasible, intermittent EN is preferred over continuous infusion, as it more closely mimics physiological feeding patterns and supports hormonal and bile acid secretion. Continuous EN may be used as an adjunct in cases of accelerated intestinal transit or intolerance. Post-pyloric feeding should be reserved for critically ill infants at high risk of aspiration or requiring frequent fasting for procedures.*

## Which milk should be provided in the pre- and post-operative period?

Appropriate nutritional management is crucial for improving outcomes in surgical newborns. A well-planned pre- and post-operative nutrition strategy can reduce morbidity and mortality, shorten hospital stays, and support better growth. However, there is no consensus on the optimal nutritional approach for these infants, resulting in considerable variability in clinical practice [41].

Preoperatively, nutrition should be optimized and growth monitored via anthropometric measurements. The American Society for Parenteral and Enteral Nutrition (ASPEN) suggests using Z-scores for weight and height, categorizing malnutrition as mild (−1 to −2), moderate (−2 to −3), or severe (≤-3) [42]. Identifying high-risk patients before surgery can help to inform post-operative nutrition strategies.

Preoperative EN should be paused as late as possible, depending on whether human milk (HM) or formula is being used: last ERAS recommendations suggest that, before surgery, for neonates who are not otherwise nothing by mouth, formula or breast milk (unfortified) could be offered for up to 6 hours and 4 hours, respectively, and clear fluids up to 1 hour to minimize fasting [43].

Post-operative feeding timing remains uncertain, but current practice favours starting EN as soon as the infant is clinically stable [43]. Early EN, typically within 8–48 hours post-surgery, is associated with shorter hospital stays, reduced infections, and a lower risk of ileus, without increasing surgical complications [44]. In abdominal surgery, continuous EN may improve absorption and tolerance, while bolus feeding favours a more physiological hormone secretion, potentially supporting motility and intestinal adaptation [45]. However, a systematic review of nine studies found no definitive advantage of either method [46]. Furthermore, the strategy for increasing feeding volumes depends on the patient’s clinical conditions and tolerance.

HM is the preferred feeding option due to its well-established benefits, including essential nutrients, immune protection, and bioactive factors promoting intestinal growth and barrier integrity [47]. The composition of HM and its microbiota play a key role in fostering a healthy intestinal microbial environment. HM should be appropriately fortified to meet the infant’s nutritional needs when necessary [48].

When mother’s own milk (MOM) is unavailable, donor human milk (DHM) is a viable alternative [49]. DHM has been shown to help infants achieve enteral autonomy faster and reduce complications associated with prolonged parenteral nutrition [30, 50–52].

In the absence of HM, there is no consensus on the best formula for post-operative feeding. American guidelines prefer hydrolysed or amino acid-based formulas; European guidelines reserve them for after standard formulas, with no clear superiority between types. No evidence exists that extensively hydrolysed formulas are superior to amino acid-based formulas for intestinal adaptation [53, 54]. Some authors suggest that formulas with intact proteins may support adaptation, especially in SBS, although increased intestinal permeability and allergy risk should be considered [55]. Amino acid-based or extensively hydrolysed formulas often lack sufficient calcium, phosphorus, and vitamin D, increasing the risk of metabolic bone disease [56]. Regular monitoring of metabolic bone markers is therefore needed. Medium-chain triglycerides in formulas can improve lipid absorption in cases of reduced intestinal capacity or cholestasis [57].

### Take home messages

1. *The panel suggests optimizing nutritional status preoperatively through growth assessment using Z-scores and initiating early EN once the infant is clinically stable, favouring HM as the primary option due to its numerous benefits for surgical newborns.*
2. *Given the lack of definitive consensus on the optimal formula when HM is unavailable, the panel suggests considering DHM as a viable alternative and highlights the ongoing debate between extensively hydrolysed/amino acid-based formulas versus standard polymeric formulas. Particular attention should be paid to the risk of metabolic bone disease when using hydrolysed or amino acid-based formulas, due to their lower content of calcium, phosphorus, and vitamin D.*

## Vitamins and trace elements in enteral nutrition

Vitamins and trace elements are essential for neonate development and biological functions across all systems. Vitamin A and zinc are crucial for cell integrity, proliferation, and differentiation. Vitamin A supports intestinal adaptation and enteric nervous system development, while zinc aids immunity and wound healing; its deficiency raises infection risk and growth retardation [58, 59].

Vitamin D and copper also have immunoregulatory roles, with vitamin D being critical for bone homeostasis [60, 61].

Selenium and vitamin E are antioxidants; deficiency in vitamin E can cause neurological damage [62, 63].

Similarly, vitamin B12 deficiency can cause myelopathies and demyelinating diseases, while vitamin K deficiency leads to coagulopathy [64].

Over 95% of vitamins and minerals absorbed enterally are taken up in the proximal small intestine, except for vitamin B12, which is absorbed in the terminal ileum. Neonates undergoing intestinal surgery are at high risk for malabsorption, with nutritional deficits depending on the extent of intestinal resection and the amount of residual intestine. Inflammation caused by bacterial growth impaired intestinal motility, and mucosal damage further contributes to malabsorption [65].

Nutritional deficiencies often occur during the transition from PN to EN and may persist after stable FEF is reached [60, 65], with 97% of children with prolonged PN dependence or less than 25% of residual small intestine experiencing deficiencies in at least one micronutrient [60].

Common deficiencies include vitamin D, zinc, and iron, with less frequent deficiencies in vitamins A, E, copper, phosphorus, and selenium [58, 60, 65].

There is limited evidence regarding appropriate micronutrient intake for neonates undergoing gastrointestinal surgery or with malabsorption. Some authors suggest doubling the normal dosage of micronutrients, although inappropriate supplementation can lead to toxicity, albeit rarely [62].

The North American Society for Pediatric Gastroenterology, Hepatology and Nutrition (NASPGHAN) and ESPGHAN provide indications for children with chronic liver disease (cholestasis) and preterm neonates weighing < 1800 grams, which can be adapted for this population (Table 1) [33, 63]. These recommendations are largely extrapolated from other specific populations and should therefore be interpreted as expert-based, provisional guidance rather than evidence-based standards. Further research is required to define optimal supplementation strategies in surgical neonates, as current evidence remains limited and population-specific data are lacking.

**Table 1:** Recommended micronutrient intake in children with chronic liver disease and in preterm infants with birth weight < 1800 g

	Children with chronic liver disease	Preterm newborns with birth weight < 1800 g
Vitamin A	< 10 Kg: 5000 UI/day	1330–3300 UI/Kg/day (400–1000 µ g retinol ester/Kg/day)
	> 10 Kg: 10,000 UI/day	
Vitamin D (cholecalciferol)	2000–5000 UI/day	400–700 UI/Kg/day ( < 1000 UI)
Vitamin E (D-α-tocopherol polyethylene glycol 1000 succinate)	15–25 UI/Kg/day	2.2–11 mg/Kg/day
Vitamin K	2–5 mg/day (1–10 mg ev or im)	4.4–28 µg/Kg/day
Iron	According to standard recommendation	2–3 mg/Kg/day (max 6)
Calcium	According to standard recommendation	3–5 mmol/Kg/day
Sodium	1–2 mEq/Kg/day	3–5 mEq/Kg/day (max 8)
Potassium	2 mEq/Kg/day	2.3–4.6 mEq/Kg/day

Adapted from Mouzaki M et al. 2019 and Embleton ND et al. 2023

In particular, supplementation with 2000 IU/day of vitamin D3 is indicated in insufficiency and 5000 IU/day in moderate to severe deficiency states [61]. 

No specific recommendations are available for zinc supplementations although parenteral doses of 500 μg/kg/day have proven effective in preventing zinc deficiency in preterm neonates and in neonates with an intestinal stoma (both preterm and term), while a dose of 250 μg/kg/day has been effective in term neonates without a stoma [66].

Vitamin B12 deficiency, linked to bowel resections and bacterial overgrowth, should be evaluated with methylmalonic acid (MMA) levels; a 200 μg intramuscular B12 dose can reduce elevated MMA [67].

Regular monitoring is needed, though timing varies from monthly to semi-annually [63, 65].

### Take home messages

1. *The panel suggests vigilant monitoring for micronutrient deficiencies in surgical newborns, particularly vitamin D, zinc, and iron, especially during the transition from PN to EN and in those with extensive bowel resection or prolonged PN dependence, adapting supplementation guidelines for cholestasis and preterm infants when specific recommendations are lacking.*
2. *Considering the high risk of malabsorption in this population, the panel suggests individualised supplementation of fat-soluble vitamins (A, D, E, K), zinc, and vitamin B12 based on the extent of intestinal resection, presence of stoma, and regular monitoring of relevant biomarkers like MMA for vitamin B12, while being cautious of potential toxicity from over-supplementation.*

## Oesophageal Atresia

OA is the most common congenital oesophageal anomaly, with 85–90% of cases involving a tracheoesophageal fistula (TOF) [68]. The incidence is between 1/2500–1/4500 live births [69]. The aetiology is multifactorial, and approximately 55% of patients with OA have other associated anomalies [68]. The diagnosis of OA is often post-natal (90% of cases) and is suspected when the nasogastric tube fails to progress into the stomach at birth. Since the first hours after birth, the newborn may present abundant salivary secretions and respiratory difficulty. Long-gap OA is defined by a gap > 3 vertebral bodies between the stumps [70].

### Preoperative nutritional management

Preoperative management involves the placement of a double-lumen suction tube in the upper stump until surgery (Replogle) with continuous suction pressure and administering total PN through a central venous catheter. The expected macro and micronutrient intakes should be personalized based on gestational age (GA) and birthweight (BW) [71]. In very selected cases, particularly when it is necessary to postpone surgical correction by weeks (or months), it is possible to proceed with the so-called “sham feeding”, an oral feeding without nutritional purposes, which may be used to promote sucking and swallowing, especially in patients with cervical oesophagostomy [72].

### Postoperative nutritional management

Postoperative management varies; some centres still use a trans-anastomotic nasogastric tube (placed during surgery), though evidence does not support reduced stenosis [71, 73]. If the trans-anastomotic tube has been placed, EN can be started after 24–48 h; a Consensus from the European Reference Network for rare Inherited and Congenital Anomalies (ERNICA) prudently proposes that an oesophagogram be routinely performed to exclude any anastomotic leaks, before starting oral feeding [71].

In the absence of leakage, the surgeon removes the trans-anastomotic tube 5–6 days after surgery. Not all centres agree on performing a postoperative oesophagogram, arguing that in case of a significant leak, the patient would present clinical signs (such as pneumothorax, pleural effusion, etc.) that would direct towards a diagnostic investigation and possible treatment [74].

No consensus exists on optimal diet or time to reach FEF in OA. Exclusive HM is preferred; DHM or formula may be used if HM is unavailable. Fortification or hypercaloric formulas (100 kcal/100 ml) may be needed for growth concerns [75].

Feeding volume can be increased up to 20 ml/kg/day, monitoring for respiratory symptoms indicating possible TOF recurrence [71].

### Management of long-gap oesophageal atresia

In cases of long-gap OA, a gastrostomy is usually placed. In clinically stable patients with a functioning gastrostomy and no postoperative complications, EN can be started through the gastrostomy as early as 3 hours after surgery, avoiding prolonged enteral fasting [76]. Surgery is typically postponed (usually after 6–8 weeks) [77].

Gastrostomy complications include local irritation, infection, leakage, and dislocation [78].

### Take home messages

1. *The panel highlights the importance of optimizing preoperative nutritional status through individualised parenteral support to maintain adequate growth before surgical repair.*
2. *The panel recommends that an oesophagogram should be prudently performed to exclude anastomotic leaks before starting enteral or oral feeding, while acknowledging the ongoing debate regarding its routine use. When an anastomotic leakage is excluded, EN* *can be started.*
3. *Exclusive HM is preferred postoperatively; when unavailable, DHM can be considered as the second option.*
4. *For long-gap OA, early EN via gastrostomy can be initiated in clinically stable patients with a functioning gastrostomy and no complications, avoiding prolonged enteral fasting.*

## Congenital diaphragmatic hernia

CDH is a congenital incomplete diaphragm closure, occurring in 1/2500–3500 births [79]. During foetal life, herniation of abdominal organs into the thorax causes pulmonary hypoplasia, requiring intubation, ventilation, and hemodynamic support at birth [80]. After stabilization, surgery repositions the viscera and closes the defect by suturing or patch placement (in larger ones) [81]. Guidelines mostly address acute and perioperative life support; nutrition is less studied. Follow-up studies have shown that feeding difficulties, malnutrition, and poor growth are significant problems [82], especially in those who need Extracorporeal Membrane Oxygenation (ECMO) or patch placement, and in those who have persistent pulmonary hypertension [83]. Nutritional and growth problems are also more frequent in newborns born preterm or requiring oxygen supplementation at discharge [84]. Extrauterine growth retardation affects nearly half of CDH infants up to 12 months [82, 85], influenced by increased respiratory effort, chylothorax, and gastroesophageal reflux [86, 87].

### Preoperative nutritional management

At birth, a nasogastric tube should be placed to decompress the stomach and the gut [88]. During the preoperative cardiovascular stabilization phase, EN is generally not indicated due to the possible worsening of respiratory failure. European recommendations recommend starting PN within 24 hours [89, 90].

To support growth in these infants, a protein intake of ≥2.3 g/kg/day [85] and a caloric intake of ≥ 120 kcal/kg/day is recommended [88–92]. However, achieving these intakes may be hindered by the clinical need to restrict fluid intake [89]. Most children with CDH present a state of “hypermetabolism” with high energy expenditure at rest (measured by indirect calorimetry), needing about 30% higher caloric intake for weight gain [93]. Therefore, compared with healthy neonates, infants with CDH require significantly higher caloric and protein intakes to sustain growth and compensate for increased metabolic demands.

### Postoperative nutritional management

Postoperatively, EN can start once bowel function recovers [89], reducing PN duration and time to FEF [94]. This transition period is particularly delicate, as it exposes newborns to the risk of protein and/or caloric deficit and consequent growth retardation [95]. Initial milk volumes ≤20 ml/kg/day are recommended, including for ECMO patients [89, 90]. Furthermore, the stool passage is crucial [89].

Due to its excellent tolerability and the benefits associated with its intake, the first-choice food is fresh HM or, if not available, DHM [96]. To maintain and increase milk production, all mothers should receive adequate support from the first days of life, and breastfeeding should be encouraged and started as soon as possible.

As a second step, formulas, including hydrolysed or amino acid-based, may be introduced to manage reflux [89]. No specific feeding method (bolus vs. continuous) shows a clear advantage; continuous or transpyloric feeding may be used if tolerance is poor or reflux risk is high. The choice of feeding modality (bolus, continuous, or transpyloric) should be individualised based on each infant’s tolerance, reflux risk, and clinical stability, as no single approach has proven universally superior. Enteral calories, necessary to obtain adequate weight gain (25–30 g/day), are generally higher than those of infants of the same age and are equal to 120–140 kcal/kg/day [89].

A delay in reaching the recommended enteral intake may be secondary to poor feeding tolerance and the presence of chylothorax or gastroesophageal reflux [88].

If volume tolerance is limited, HM fortifiers or high-calorie formulas may help to meet needs [88].

According to tolerance, milk supply increases should be gradual to prevent gastric overdistension and reduce the risk of reflux. An increase of 20 ml/kg/day or even higher quantities may be well tolerated, especially in cases of transpyloric tube feeding or when intragastric supply has been well tolerated [88].

Growth (weight, length, head circumference) should be monitored weekly [88]. Antacid therapy should be reserved for selected cases [94], after careful risk–benefit evaluation [89].

Internal nutritional protocols may reduce PN duration, improve growth, and lower complications, as seen in other neonatal populations [97].

Some children cannot be fed orally, often due to the development of an oral aversion. To achieve an adequate caloric/protein intake, some neonates (up to 42% according to some authors) may require long-term exclusive or supplementary feeding via a nasogastric tube [98], and for some (up to 29% of cases), it may be necessary to create a gastrostomy during hospitalization or after discharge [99].

Delayed oral feeding and gastrostomy are more common in severe cases, large defects, prolonged ventilation, ECMO, or chromosomal abnormalities [100].

### Take home messages

1. *The panel suggests starting PN as soon as possible, given the complex hemodynamic instability and the initial contraindication to EN before surgery.*
2. *After surgical repair, EN should begin as soon as bowel function is restored, preferably with HM; when unavailable, DHM or formulas should be used to meet nutritional needs.*
3. *Infants with CDH require higher caloric and protein intakes than healthy neonates due to hypermetabolism, and careful growth monitoring is essential, considering that malnutrition and feeding difficulties are common in infants with CDH.*
4. *The choice of feeding modality (bolus, continuous, or transpyloric) should be individualised according to tolerance and reflux risk, as no single method has proven superior.*

## Chylothorax

Chylothorax is the most common neonatal pleural effusion, caused by lymph leakage into one or both pleural cavities. The diagnosis is confirmed when the pleural fluid analysis shows more than 70–80% lymphocytes and, in enterally fed patients, a triglyceride concentration greater than 110 mg/dl (1.1 mmol/L) [101].

Congenital form is a rare condition (1/10000–1/24000 live births) and is linked to lymphatic flow disorders, often in syndromes like Down, Turner, Noonan, or Ehlers-Danlos [101, 102]. Foetal chylothorax can impair lung development, cause hypoplasia, heart failure, and hydrops [103].

Conversely, acquired forms are usually postoperative complications (e.g., cardiac/thoracic surgery) [101].

Lymph contains lymphocytes, proteins, fats, electrolytes, vitamins, and immune factors. Losses can lead to malnutrition, immune suppression, coagulopathy, and infection risk [102].

Nutrition aims to reduce lymph flow while supporting growth and development. Options include withholding EN, using PN, or using a formula containing medium-chain triglycerides as the lipid source, or defatted and fortified HM. To safeguard MOM, low-temperature centrifugation (at 3600 rpm for 20 min at a temperature of 5 °C) proved to be very efficient in defatting HM, reducing the concentration of triglycerides by 85% and cholesterol by 50%. Macronutrient content (proteins, albumin, and immunoglobulin A) did not change significantly due to defatting and pasteurization procedures [104].

Strong evidence to choose one or the other option is lacking, but we suggest considering fasting and PN for 5–7 days in high-flow ( > 10 ml/Kg/day) chylothorax allowing time for drainage to reduce, while EN can be started in low-flow (≤10 ml/Kg/day) and asymptomatic cases [101]. These management strategies are based on expert consensus and clinical experience rather than high-level evidence, and should therefore be adapted to the individual infant’s clinical condition and response.

Octreotide, a somatostatin analogue, is the most commonly used medication to decrease the volume of chylous effusion. It likely reduces lymph flow by splanchnic vasoconstriction and lower secretions. Despite some investigators describing possible benefits [105], evidence is limited, and Cochrane reviews don’t confirm its efficacy [106].

We suggest considering octreotide when the initial nutritional strategy is ineffective in reducing the volume of chylous effusion. Starting at minimum dosage (1–2 mcg/Kg/h), titrating up to a clinical response with a maximum dose of 10 mcg/kg/h. Duration of therapy commonly ranges from 7 to 14 days [102]. Octreotide can be given subcutaneously if intravenous access is lost. Given the paucity of robust evidence, these recommendations should be considered as expert-based, and individualised according to clinical response and tolerance.

The treatment goal is to decrease the permanence of the chest tube and expedite return to a regular diet. We suggest removing the chest tube when drainage is ≤ 2 mL/Kg/day without medication.

To minimize complications related to high-flow or long-lasting chylothorax, we suggest to strictly monitor but to replace albumin (albumin 20% 1 g/kg/dose) only in case of hypoalbuminemia and oedema/lymphoedema (third spacing) and intravenous immunoglobulins (400 mg/Kg) only in case of hypogammaglobulinemia and sepsis, because the correction may worsen chest tube drainage introducing a considerable fluid burden. Coagulation parameters should be checked to monitor for the development of coagulopathy [102].

While the baby is on a fat-modified diet, essential fatty acids and fat-soluble vitamins should be regularly replaced (20% lipid solution, 3 g/kg weekly, or 1 g/kg three times a week).

The duration of a fat-modified diet after the resolution of chylothorax is controversial. While 4–6 weeks is traditionally prescribed, some report no recurrence with only 2–3 weeks of a fat-modified diet [107]. We suggest a 2–3 week fat-modified diet after chest tube removal.

In refractory chylothorax unresponsive to conservative therapy (when drainage persists at least 3–4 weeks after diagnosis despite dietary modification and/or medications or is such high output to be life-threatening), secondary surgical interventions may be required to attain resolution [101, 108].

### Take home messages

1. *The panel suggests enteral fasting and PN for 5-7 days in high-flow ( > 10 ml/Kg/day) chylothorax, followed by the introduction of fat-modified EN* *using MCT-based formulas or defatted HM when drainage decreases below 10 ml/kg/day.*
2. *The panel suggests adding octreotide when nutritional management alone is ineffective; dosing and duration should be individualised and are based on expert consensus given the limited evidence.*
3. *Replacement therapies such as albumin, intravenous immunoglobulin, fat-soluble vitamins, and essential fatty acids should be considered in selected cases to prevent or correct nutritional and immune deficiencies.*
4. *The panel suggests chest tube removal when drainage is ≤ 2 mL/Kg/day without medication, with a 2-3 week fat-modified diet after chest tube removal.*

## Intestinal atresia

Intestinal atresia is a congenital malformation characterized by a discontinuity of the lumen between the duodenum and the rectum. Fasting and total PN are always required before and after resection surgery and intestinal anastomosis. Feeding strategies depend on prematurity, weight, comorbidities, and residual bowel length. Postoperative hypomotility, especially in the proximal tract, may delay feeding and prolong hospitalization [109].

Some studies suggest early feeding may reduce infections and hospital stay without increasing dehiscence. Minimal enteral feeding (MEF) may improve tolerance and reduce bacterial translocation [20, 110].

A recent multicentre RCT found no difference between early (within 48 h since surgery) and delayed feeding (after biliary stasis < 15 mL/kg/day). Early feeding is safe but doesn’t significantly impact outcomes (length of stay and postoperative complications) [44]. Despite these findings, most centers still delay enteral feeding by 5–10 days, especially in proximal atresia, due to concerns about postoperative hypomotility, gastric stasis, and feeding intolerance. This reflects a cautious, experience-based approach that often diverges from trial protocols, balancing theoretical safety with clinical practicality.

Generally, reduction of biliary stasis, presence of bowel movement, and minimal abdominal and respiratory involvement are the variables that indicate the possibility of starting feedings. This typically occurs around 5–6 days postop for distal atresia, and around 7–10 days postop for proximal atresia.

Feeding can begin with volumes of about 10 ml/kg/day, gradually increased according to feeding tolerance. The type of malformation (type III b and IV atresia) and the level of obstruction may affect the timing and increase of EN, especially in cases of SBS.

In neonates who underwent surgery for duodenal or intestinal atresia types I, II, and IIIa, where the resection is generally limited to the atresic segment, breastfeeding (exclusive or combined with formula milk) is almost always possible, and even strongly recommended to reduce the risk of NEC.

In types IIIb and IV, extensive resection often leads to SBS, requiring prolonged PN and bowel adaptation [111]. In these infants, HM plays a fundamental role in promoting intestinal adaptation and recovery, thanks to its trophic and anti-inflammatory properties, bioactive components, and ability to support a healthier microbiota. When unavailable, DHM should be the preferred alternative.

### Take home messages

1. *The panel supports early EN following intestinal anastomosis in neonates with intestinal atresia, recognizing that although RCTs demonstrate its safety, many centers still delay feeding (particularly in proximal atresia) because of concerns regarding postoperative motility and tolerance.*
2. *EN should be started at 10 mL/Kg/day and increased by standard feeding progression, according to feeding tolerance.*
3. *HM should be the preferred feeding option after surgery for intestinal atresia, as it promotes intestinal adaptation and reduces the risk of complications.*

## Abdominal wall defects

Omphalocele and gastroschisis are congenital abnormalities involving the abdominal wall, leading to herniation of internal organs. While both conditions share some embryological similarities, they differ in clinical manifestations [112], needing distinct approaches [113, 114]. Nutritional management is a critical aspect of the care for neonates with these defects, aiming to support intestinal development and recovery. A standardised feeding protocol is essential for managing neonates with abdominal wall defects, starting PN within 24 hours of life and then introducing EN within 7–12 days, with FEF tolerance expected by day 23 [115]. EN should be advanced according to BW, intestinal motility, and gut length. However, these protocols should be regarded as structured frameworks rather than rigid standards, as feeding initiation and advancement must be individualised based on the specific defect (omphalocele vs simple or complex gastroschisis), bowel function, and clinical stability.

Whenever possible, HM is preferred for gut growth and immune support [116]. HM remains the feeding option of choice in both conditions, as it promotes intestinal adaptation, reduces the risk of infection and cholestasis, and supports overall growth; DHM should be used when MOM is unavailable.

### Omphalocele

Omphalocele occurs due to incomplete closure of the abdominal wall, often leading to a worse prognosis due to associated congenital anomalies, particularly cardiac defects (e.g., tetralogy of Fallot) and chromosomal disorders (e.g., trisomies 13, 18, 21) [112]. The nutritional approach depends on the size of the omphalocele and the neonate’s clinical condition:

- Preoperative period: Total PN is usually initiated while waiting for the intestine to resume its function. Once intestinal function is adequate, EN may begin, especially in conservatively managed cases.
- Postoperative period: After surgical correction, total PN is maintained until intestinal function resumes, gradually transitioning to oral feeding. Regularly monitoring feeding tolerance is essential, and adjustments are made based on the infant’s response [117, 118].

### Gastroschisis

Gastroschisis involves exposed intestines through a wall defect. Amniotic fluid exposure can cause malrotation, atresia, or SBS. Multidisciplinary care is essential due to impaired motility and malabsorption. Surgery to correct gastroschisis may further complicate motility due to ischemic damage [113–115]. The Gastroschisis Prognostic Score is a score based on bowel appearance after birth (visual scoring of bowel matting, necrosis, atresia, and perforation), able to predict SBS and guide management [119, 120].

- Simple gastroschisis: These cases typically require a short duration of PN, with early initiation of EN (around 7 days of life) and the goal to reduce hospital stay. The average duration of PN is about 3 weeks, with discharge occurring around weeks 4-5.
- Complex gastroschisis: These cases require longer periods of PN and are associated with higher risks of infection and cholestasis. The cycling of PN is suggested to manage these risks [121–123].

### Take home messages

1. *The panel highlights that standardised feeding protocols provide a useful framework but must be individualised according to the type of abdominal wall defect, intestinal motility, and clinical stability.*
2. *PN should be initiated within the first 24 hours of life to ensure adequate nutritional support while intestinal function develops.*
3. *HM (MOM or DHM) is the preferred option for enteral feeding in both omphalocele and gastroschisis, as it supports intestinal growth, immune protection, and adaptation.*
4. *Simple and complex gastroschisis require distinct nutritional strategies: simple cases generally allow earlier EN and shorter PN duration, whereas complex cases often need prolonged PN.*
5. *The Gastroschisis Prognostic Score, based on bowel appearance at birth, is a valuable tool to stratify risk, predict outcomes such as SBS, and tailor nutritional management accordingly.*

## Hirschsprung Disease

Hirschsprung’s Disease (HD) is a congenital absence of ganglion cells in the distal bowel, extending proximally from the internal anal sphincter [124]. It presents with delayed meconium, distension, GI intolerance, and bilious vomiting [125]. The cause is failed neural crest cell migration; earlier arrest leads to longer aganglionic segments [126]. The incidence of HD is approximately 1:5000 live births, with a male-to-female ratio of 4:1 [127, 128].

About 20% have associated anomalies [129] Down syndrome is the most common [130].

Management is both medical and surgical, with goals of emptying, preserving function, and continence. Medical options include diet, behaviour changes, meds, and enemas to facilitate colon emptying [131].

Surgery removes or bypasses the aganglionic segment via pull-through or ostomy. The three most used procedures are Swenson and Bill’s rectosigmoidectomy, Duhamel’s retrorectal approach, and Soave’s endorectal pull-through procedure [124].

HM is the preferred choice in newborns affected by HD. In the absence of HM, consideration may be given to the choice of constipating diets in the case of excessive faecal leakage, or soluble fibres such as pectin in cases of constipation. Among the medications available for paediatric use are osmotic laxatives, such as polyethylene glycol, lactulose, or magnesium milk [132].

Both before and after surgery, it is necessary for HD patients to be regularly followed at a specialized centre to monitor their nutritional status and bowel habits [127].

Complications include infections, leaks, adhesions, and long-term constipation (often from residual aganglionosis or high anal tone) [133].

Use of osmotic laxatives and repeated contact between stools and skin can cause postoperative skin lesions and dermatitis, both in the perianal area and, in the case of enterostomy patients, in the peristomal area. For this reason, the use of barrier creams is recommended until bowel emptying slows down [134].

Hirschsprung-associated enterocolitis (HAEC) is a major complication, occurring in 20–58% of cases, even after surgery. Soiling affects 10–30% of patients post-pull-through. Achieving normal post-operative bowel function clearly depends on the intensity of bowel training, the social background, and any cognitive deficit of the patient [135].

### Take home messages

1. *The panel suggests that HM should be the primary source of nutrition for newborns with HD, due to its beneficial effects on feeding tolerance, intestinal function, and overall development.*
2. *In infants with HD experiencing excessive fecal leakage, a constipating diet may be considered, whereas those with constipation may benefit from soluble fibres such as pectin, under professional supervision.*
3. *Osmotic laxatives (polyethylene glycol, lactulose, magnesium milk) may be used to facilitate bowel emptying when dietary adjustments alone are insufficient.*
4. *Regular long-term follow-up at specialized centres is essential to monitor bowel function, nutritional status, and growth, and to guide ongoing management.*
5. *Skin protection strategies, including the use of barrier creams, should be implemented to prevent or treat perianal and peristomal lesions resulting from frequent stool contact.*

## Necrotizing enterocolitis: preventive strategies

NEC is an inflammatory disease leading to intestinal necrosis. The incidence of NEC is approximately 6% in very low birth weight (VLBW) infants, and 8% in infants with BW < 1000 grams (ELBW - extremely low BW) [136, 137]. NEC is burdened by high mortality and long-term morbidity [137–139]: prevention is a priority, through the implementation of nutritional and non-nutritional interventions [140].

- HM: Raw HM is preferred [141]; if unavailable, DHM is the recommended alternative [142]. Feeding HM reduces the risk of NEC compared to cow’s milk protein-based formula [143–147] by improving gut pH, motility and microbiota via its bioactive components [148–150]; its protective effect is dose-dependent [151].
- HM fortification (HMF): it helps prevent extrauterine growth restriction and nutritional deficits, without increasing NEC risk [152], and is recommended for all infants < 1800 grams [33, 48]. HMF allows the addition of proteins, energy, minerals, trace elements, vitamins, and electrolytes to meet the nutritional needs of preterm infants.

HMF could begin with standard fortification (adding a fixed amount of multicomponent fortifier per 100 ml), and if the infant’s growth remains inadequate, it could be switched to individualised fortification [153]. Individualised fortification can be targeted or adjustable. Targeted fortification is based on the analysis of HM and the addition of macronutrients to the standard fortification to reach the recommended intakes [154, 155]. Adjustable fortification consists of adding a multicomponent fortifier plus protein supplementation based on the blood urea nitrogen value [156]. Both modalities are valid depending on the individual experience and expertise of the centre.

- Standardised Feeding Protocols: They are associated with lower incidences of NEC [157], by maintaining a linear rate of advancement and avoiding potentially harmful periods of acceleration or deceleration, adopting three cornerstones: a) starting MEF (≤24 ml/kg/day) within 96 hours of life reduces NEC risk [158–160]; b) delaying EN beyond 96 hours doesn’t reduce NEC but delays full enteral feeds [161]; c) advancing feeds at 30–40 ml/kg/day is safe in most cases, but slower progression is advised in high-risk infants (e.g., ELBW or those with prenatal Doppler alterations, including absent or reverse end diastolic flow in the umbilical arteries), where the currently available evidence is not sufficient to establish the safety of fast EN progression and thus slow progression is recommended [162–164].
- Oral colostrum therapy (OCT): It consists of administering drops of maternal colostrum into the mouth of the newborn within the first day of life, which are mainly directly absorbed by the oral mucosa. A RCT found that OCT (≤0.4 ml) started in the first 48 h may reduce NEC, sepsis, intraventricular hemorrhage, and time to full feeds in infants ≤32 weeks GA [165], but a systematic review before this trial was inconclusive regarding the benefit of OCT concerning NEC, probably due to the low incidence of NEC in the considered studies [166]. Pending further RCTs, the currently available evidence suggests the possible use of OCT in neonates with GA ≤ 32 weeks.
- Withholding enteral feeding during red blood cell transfusion: An association between red blood cell transfusion and the development of NEC (TRAGI - transfusion-associated acute gut injury) has been reported in preterm infants [167–169]. TRAGI may occur within 48 h post-transfusion, and the exact pathogenetic mechanism, as is the role of EN, is not yet known. It would seem that a role is played by intestinal hypoxic suffering resulting from anaemia [170–173]. Although the association between transfusion and NEC has been questioned [174],

pending further evidence, cautious feeding during transfusions is advised in high-risk infants (GA < 30 weeks, BW < 1500 g, haematocrit ≤25%, haemoglobin ≤ 8 g/dL) [173].

- Enteral Nutrition and Treatment of Patent Ductus Arteriosus: The lack of evidence for or against EN during pharmacological therapy of persistent ductus arteriosus prevents any recommendation [175]. Pending definitive evidence, a cautious approach using MEF with HM is reasonable during PDA treatment [176].
- Probiotics: Dysbiosis is a risk factor for NEC [177–179]. A recent systematic review and network meta-analysis, including 106 clinical trials and 25,840 preterm infants, found that multi-strain probiotics were associated with reductions in all-cause mortality, NEC, feeding intolerance, and hospitalization [180]. ESPGHAN has expressed its support for the use of probiotic strains with proven efficacy, since their use is associated with a reduction in the incidence of sepsis of 30-50% [181].

### Take home messages

1. *The panel suggests using HM as the main strategy to prevent NEC; HMF is recommended for all infants with a birth weight < 1800 grams.*
2. *The panel suggests that each neonatal intensive care unit adopt standardised feeding protocols that regulate the initiation and progression of EN and establish agreed-upon feeding rules during blood transfusion and treatment of patent ductus arteriosus.*
3. *The panel suggests using probiotics as a preventive strategy against NEC.*

## Necrotizing enterocolitis: nutrition during and after the acute phase

### Parenteral Nutrition

PN supports intestinal rest and supplies essential nutrients in NEC [182]. Guidelines recommend adjusting PN energy intake by illness phase: 45–55 kcal/kg/day (early acute), 60–65 (late acute), and 90–120 (recovery) [15].

Proteins are essential during NEC to support tissue repair [123], while strict biochemical monitoring [124] helps avoid under- and overnutrition. Stress-induced insulin resistance often causes hyperglycaemia, worsening outcomes [183]. Multiorgan failure with hypoperfusion and lactic acidosis raises the risk of electrolyte imbalances, especially hyponatremia and hyperkalaemia [10]. Thus, strict monitoring of fluids, electrolytes, and nutrient tolerance is essential [184, 185].

### Enteral Nutrition

- **What:** HM (MOM or DHM) remains the preferred option after surgical NEC [1, 5, 26, 45, 184]. If unavailable, preterm formula is recommended [26, 184], for its higher caloric density and optimized composition (lower lactose, higher medium-chain triglyceride and long-chain triglycerides contents). Semi-elemental or elemental formulas are suggested in case of intolerance [26].
- **When:** In stable infants, EN may begin 5–7 days post-NEC without increasing complications [186–190].
- **How:** After an episode of NEC, several authors suggest starting with MEF and then increasing by 10-20 ml/kg/day [31, 184, 191], while in case of intestinal resection or in ELBW, a more cautious regimen (increase by 1 ml every 24 hours) was suggested [192]. No clear advantage exists between bolus and continuous feeding [33], though bolus is more physiologic [193]. Continuous or combined regimens may benefit neonates with SBS [32, 194]. A practical approach could be to start MEF with HM (bolus or continuous), adding small volumes of oral milk as soon as possible. Standardised Feeding Protocols post-NEC reduce time to 50% EN and lower incidence of intestinal failure-associated liver disease (IFALD) [195, 196].

### Take home messages

1. *The panel suggests administering PN to provide different macro and micronutrient intakes according to the phase of the newborn’s illness.*
2. *The panel suggests the use of HM as the primary strategy also for restarting EN after an episode of NEC; whenever possible (clinical and radiological signs) MEF should be started between 5 and 7 days after the acute episode with the enteral progression at standard feeding progression, according to tolerance. Preterm/term formulas are recommended when HM is not available.*
3. *When oral feeding is not possible, a practical approach could be to start with bolus or continuous feeding and provide some volume of oral HM as soon as possible.*

## Enterostomy Care

Neonates may require enterostomy due to congenital or acquired intestinal diseases. Significant bowel exclusion often necessitates prolonged PN. These infants are at risk of dehydration, electrolyte imbalance, and growth failure during EN advancement [197, 198]. Additional risks include IFALD, catheter-related infections, and neurodevelopmental delay [199, 200].

Three enterostomy types exist:

1. End stoma without mucus fistula: the proximal bowel end is brought out through the abdominal wall as a stoma, and the distal end is closed and left within the abdominal cavity.
2. Stoma with mucus fistula: the bowel is interrupted, and both ends are brought out through the abdominal wall. The proximal end is the functioning stoma and the distal end is the mucus fistula.
3. Loop stoma: This is formed with the bowel in continuity by suturing a bowel loop to the abdominal wall and making an opening into the bowel.

High enterostomies (HEs) are located at the jejunum or proximal ileum level, while low enterostomies (LEs) are located at the distal ileum and colon level. HEs are associated with a greater risk of dehydration [201].

HEs have high stomal outputs (SO). Some authors define high SO when > 20 mL/Kg/day for term infants and > 30 mL/Kg/day for preterm infants; other authors define high SO when > 40 mL/Kg/day for VLBW infants [192, 202]. These thresholds vary among studies because of differences in patient populations and measurement methods. Therefore, they should be viewed as general reference ranges rather than strict diagnostic cut-offs, highlighting the need for clinical judgment and individualised interpretation.

Generally, SO ≤ 20 mL/Kg reveals LEs, while SO > 20 mL/Kg/day identifies HEs [203]. Since the residual bowel absorption depends not only on its length but also on its maturity and functional capacity, LEs can have high OS, especially in the immediate post-surgical period. Other causes of high OS are intestinal infections and bacterial overgrowth syndrome.

Monitoring of infants with enterostomy should include:

1. Daily weight and weight trends that can reveal dehydration, insufficient nutritional intake, malabsorption, and electrolyte abnormalities [204]
2. Daily fluid balance, which should also take SO into account;
3. Weekly urinary sodium (uNa) is a more reliable indicator of natremia to monitor the residual bowel’s absorption capacity and sodium deficiency. uNa excretion reflects dietary sodium intake: low sodium intake activates the aldosterone-angiotensin-renin system, leading to increased aldosterone production and renal sodium reabsorption.

Infants with enterostomy may have poor intestinal sodium absorption, resulting in activation of the aldosterone-angiotensin-renin system and increased renal sodium absorption [205]. uNa ≥20 mmol/L indicates adequate sodium intake and absorption; uNa < 20 mmol/L indicates sodium deficiency and the need to increase sodium supplementation [26].

HM is the first choice post-enterostomy; if unavailable, DHM is the second choice [1], and formulas the third one [206]. If formulas are not well tolerated, as demonstrated by a persistent high SO that does not allow EN progression, extensively hydrolysed formulas may be considered; amino acid-based formulas should be reserved in case of allergy to milk proteins [26, 206, 207].

No trials define EN timing and advancement, but early EN ( < 7 days) is considered safe [189, 190]. Most centres start EN with canalization of the stoma [208]. GR, in the presence of a productive stoma, is unreliable for assessing EN tolerance, since gastric hypersecretion is part of the intestinal adaptation process after intestinal resection [209]. EN should start as early as possible, no later than 7 days after surgery, with a quantity of milk < 20 mL/kg [206].

When SO is ≤ 20 mL/Kg, EN can be increased by 10–20 mL/Kg/day, based on feeding tolerance. When SO is > 20 ml/Kg, in order to avoid dehydration during EN progression, the amount of SO and diuresis, and the presence of peristomal lesions should be considered, as proposed by Groselin et al. [32]:

- If SO 20-48 mL/Kg/day, diuresis > 2 mL/Kg/h, and absence of peristomal lesions, EN can be increased by 10 mL/Kg/day.
- If SO 48-72 mL/Kg/day, diuresis > 2 mL/Kg/h, and the presence of minimal peristomal lesions, a daily increase of EN should be avoided.
- If SO > 72 mL/Kg/day, diuresis < 2 mL/Kg/h, and the presence of peristomal lesions, EN should be reduced by 10 mL/Kg/day.

In the case of high SO enterostomy, mucous fistula refeeding (MFR) can be considered. It involves the introduction of the effluent from the stoma into the mucous fistula in order to reinhabit the excluded bowel while waiting for recanalization [201]. Before MFR, a radiological evaluation to assess the patency of the excluded bowel should be performed after at least 4–6 weeks from the acute event in the case of NEC [201, 210].

MFR appears to be associated with a shorter duration of PN, a lower incidence of cholestasis, a more rapid achievement of total EN after anastomosis, and a shorter hospital stay [211]. The MFR procedure requires caution and asepsis to avoid complications such as bleeding, intestinal perforation, and infection. Faecal material produced no more than 4 hours before the procedure should be recycled with a slow infusion (1 mL/min) to minimize the risk of dumping syndrome.

### Take home messages

1. *The panel suggests monitoring fluid and electrolyte balance, especially urinary sodium, in infants with enterostomy, and interpreting stomal output values in the context of gestational age, birth weight, and overall clinical status.*
2. *The panel suggests the use of HM as the primary strategy for restarting EN infants with enterostomy; EN should start no later than 7 days after surgery, and EN should be increased according to feeding tolerance, the amount of SO, diuresis, and the presence of peristomal lesions.*
3. *In the case of high SO enterostomy, MFR can be considered.*

## Nutritional management of infants affected by intestinal failure

Intestinal failure (IF) is a rare condition where bowel function or mass is insufficient to absorb nutrients and fluids needed for survival and development [212]. SBS is the leading cause of IF in infants (24.5/100000 live births), with an incidence that is inversely related to BW [213]. NEC is the main cause of SBS in newborns, especially in VLBW, followed by conditions such intestinal atresia, gastroschisis, and volvulus [214]. SBS severity depends on length of residual bowel, with additional factors such as the site of resection, the quality of residual bowel, the presence of the ileo-caecal valve, the length of remaining colon and the presence of IFALD modulating outcome [215].

PN has improved outcomes in IF, contributing to improve growth and clinical outcomes [216], although early EN is essential for adaptation. The intestinal adaptation process includes functional and structural changes that occur after intestinal resection, and it could last for years. Scoring systems predict PN dependence and chance of achieving enteral autonomy [215, 217–219].

### Enteral Nutrition in intestinal failure

EN should be started as soon as possible and gradually increased according to tolerance. It may be useful to use a continuous EN method, at least overnight, because it could be associated with greater benefits in terms of nutrient absorption [217]. In turn, split meals may enhance hormonal stimulation of adaptation [220].

The maintenance of eating skills (sucking and swallowing), even with minimal volumes of milk, helps to prevent food aversion [221].

Fresh MOM is preferred for its bioactive components (growth factors, hormones, nucleotides, and immunoglobulins) that support adaptation and immunity [222]. No consensus exists on alternative milk when MOM is unavailable and whereas the use of pasteurized is not possible [192]. Increasing evidence suggests the use of standard formulas with whole proteins that could be useful in promoting intestinal adaptation; however, hydrolysed protein formulas could be more tolerated than those with whole proteins and contain a greater amount of medium-chain fatty acids and a lower quantity of lactose. Some authors suggest starting with elementary amino acid formulas, both to promote absorption and because these newborns are at greater risk of eosinophilic gastroenteropathies and allergy/intolerance to cow’s milk proteins [222, 223]. Formula selection should follow a stepwise approach: standard whole-protein formulas are preferred when HM is unavailable, hydrolysed formulas may be used in cases of moderate intolerance or malabsorption, and amino acid–based formulas should be reserved for severe malabsorption or confirmed milk protein allergy.

### Cycling of parenteral nutrition and discharge criteria of these infants

Cycling of PN refers the administration of PN in a variable period of time less than 24 hours to improve the mobilization of infants with IF and their quality of life [223]. The cycling of PN reduces the risk of metabolic consequences such as hepatobiliary damage. To proceed with the cycling of PN, the patient needs to tolerate EN during the hours free from PN. According to the metabolic balance (especially in terms of glucose homeostasis), the PN-free interval can be progressively increased until the administration of PN is obtained only overnight.

ESPGHAN guidelines for managing home PN consider infants with expected dependence on PN for at least three months [26] and discharge criteria are the following: stable conditions (metabolic stability, stable growth, gastrointestinal tolerance); presence of a long-lasting and stable central venous catheter (CVC); cycling of PN; adequate and verified training of at least two caregivers who are able to take care of the connection and disconnection of PN in asepsis, carry out the dressing of the CVC, and recognize the early signs of a possible complication; presence of tools and materials at home (dedicated refrigerator, bags, infusion sets, infusion pumps, sterile kits); contact with local health facilities.

Once the stability criteria have been reached, the ESPGHAN guidelines recommend a pre-discharge meeting with the presence of the family and all those involved in the patient’s care. Indeed, in addition to the staff of centre specialized in home artificial nutrition, the management of a patient with IF involves a multidisciplinary team consisting of paediatric gastroenterologist, paediatric surgeon, neonatologist/paediatrician, nurse, dietician, pharmacist and social worker; the presence of other support figures such as radiologist, speech therapist, infectious disease specialist, vascular surgeon and local health facilities is also fundamental [224].

### Follow-up and complications

The follow-up of infants with IF aims to promote intestinal adaptation, ensure adequate growth and quality of life, and prevent the onset of possible mechanical and infectious complications. In particular, bloodstream infections are of particular concern [225]. Fever can be a sign of these infections and can occur at the start of PN administration. These complications require early clinical evaluation and blood culture from CVC, peripheral blood, and early antibiotic therapy [214].

Metabolic complications related to PN administration, especially those with immediate or early onset (within 24–48 hours), are linked to the quality and quantity of nutrients. Electrolyte alterations, hyperglycaemia, glycosuria, and refeeding syndrome can arise both in the initial phase and during changes to the mixtures. For these reasons, close clinical monitoring is mandatory [226]. On the other hand, IFALD has a multifactorial onset, associated with the prolonged use of soybean oil-based lipid emulsions [207]. In the last years, the incidence of IFALD has been reduced due to the use of composite lipid mixtures, containing fish oil, the reduction of the intravenous lipid quota, and the cyclization of the PN with the presence of time intervals free from the administration of lipids [207].

Furthermore, an early EN allows the contraction of the gallbladder and avoids stasis. Treatment with metronidazole, ursodeoxycholic acid, phenobarbital, or cholecystokinin may be helpful in some patients with cholestasis [227].

One of the main complications associated with the refeeding phase and weaning from PN in IF is intestinal dysbiosis linked to impaired bacterial growth in the small intestine (SIBO: small intestine bacterial overgrowth) [207]. The first clinical manifestations of this complication are meteorism and abdominal distension, symptoms of protein and vitamin malabsorption, poor growth, or worsening of any liver disease. It is caused by excessive bacterial growth in dilated intestinal areas characterized by impaired motility. The loss of the ileocecal valve, prolonged therapy with proton pump inhibitors, and the presence of dilated residual loops with poor motility may be further factors predisposing to excessive intestinal bacterial growth in the small intestine. In addition to a malabsorption syndrome, this can also result in bacterial translocation and D-lactic acidosis, which are linked to the bacterial production of D-lactic acid. Treatment of SIBO involves the use of antibiotics and probiotics. In some cases, it may be useful to start periodic cycles of decontaminating antibiotic therapy in order to reduce the risk of sepsis, but this therapeutic approach is not universally shared, as it could contribute to the onset of bacterial resistance [228].

Among the complications associated with the intestinal adaptation phase, it is necessary to include perianastomotic ulcers, which may be linked to intestinal dysbiosis and the dilation of the loops during the adaptation phase. They typically manifest with haematochezia (occult or overt) and progressive anaemia. This complication can occur even many years after weaning from PN, which is why long-term follow-up is mandatory in patients with IF [229].

In Table 2, we described the timepoints of clinical evaluations and diagnostic tests that we usually schedule [214].

**Table 2:** Clinical and diagnostic follow-up for infants with IF

Times	Clinical evaluations	Diagnostic tests
1–3 months	Anthropometric measurements Medical examination Dietetic review	• Blood cell count, coagulation • Liver function, electrolytes • Renal function • Blood gas analysis
6–12 months	Anthropometric measurements Medical examination Dietetic review	• Dosage of vitamin A, D, E, B12 • Iron balance • Abdominal ultrasound • Body composition • Bone density scan
12 months	Anthropometric measurements Medical examination Dietetic review	• Chest x-ray (check of central venous catheter position) • Echocardiography • Vessels Ultrasound

### Surgical therapy

When intestinal dilation is observed, peristalsis is less effective in this tract, where antiperistaltic movements can occur. Over time, this mechanism can cause SIBO, mucosal alterations, and ulcers, especially in the pre-anastomotic site. To promote intestinal lengthening by reducing this complication, various surgical techniques have been used with the aim of decreasing the diameter of the dilated intestinal lumen and promoting peristalsis (Tapering) or lengthening it (STEP or LILT procedures):

- the Tapering technique consists of a longitudinal enteroplasty by sectioning the full-thickness intestinal wall or only the muscle serosa layer. It has the disadvantage of reducing the intestinal surface;
- the STEP (Serial Transverse Enteroplasty Procedure) technique uses oblique stapled cuts to narrow and lengthen the intestine. It is simple, preserves blood supply, and avoids enterotomies. The main disadvantage concerns the impossibility of a new lengthening operation in the same intestinal tract affected by the procedure.
- the LILT (Longitudinal intestinal lengthening and tailoring) technique involves doubling the length of an intestinal segment at the expense of the diameter. This technique has the disadvantage of not increasing the absorbent mucosal surface but reducing the intestinal calibre improves its functionality.

These surgical techniques have improved the outcome of the IF, contributing, in some cases, to weaning from PN [230].

### Take home messages

1. *The panel suggests introducing early EN in infants with IF to promote intestinal adaptation, even in minimal volumes, while continuing PN as needed.*
2. *HM is the preferred option, but when unavailable, standard whole-protein formulas should be used to stimulate adaptation; hydrolysed formulas may be adopted in cases of poor tolerance, and amino acid–based formulas reserved for severe malabsorption or allergy.*
3. *The panel suggests a multidisciplinary approach for managing infants with IF, emphasizing timely initiation and gradual advancement of EN, considering HM or appropriate formulas, and a structured process for PN cycling and eventual discharge based on established criteria.*
4. *The panel highlights the importance of long-term follow-up for infants with IF due to potential complications such as bloodstream infections, metabolic disturbances, IFALD, SIBO, and perianastomotic ulcers, also noting that surgical interventions can improve outcomes in some cases.*

## Oral feeding in neonates with gastrointestinal surgical disorders

Oral feeding is a complex ability that develops from around 10–11 weeks of gestation and continues after birth [231]. Surgical neonates often face delayed oral feeding due to their condition, surgical procedures, and prolonged fasting periods; prematurity or low BW can further delay progress [3]. Once gut function resumes, EN should be restarted orally, as this stimulates trophic factors like salivary epidermal growth factor and helps prevent feeding intolerance [26]. The decision about when to start oral feeding should be made following a standardised evaluation of the neonate’s oral abilities. For this purpose, scoring systems developed initially for preterm infants can also be used to assess oral feeding readiness in the surgical population: one useful score in this respect is the 18-item Preterm Oral Feeding Readiness Assessment Scale (POFRAS) [232, 233]. The development of neonatal oro-motor skills should be promoted through active strategies: besides non-nutritive sucking and oral colostrum administration, which might reduce time to FEF, NEC and late-onset sepsis [234, 235], research shows that structured interventions (such as the Premature Infant Oral Motor Intervention, better known as PIOMI) can promote oro-motor skills, reduce time to full feeds, and improve outcomes [236]. Though designed for preterms, these interventions may benefit surgical neonates as they don’t interfere with fasting. Little evidence is available about promoting oral feeding in neonates with surgical gastrointestinal disorders: a pilot study by Tucker et al. tested “sham feeding” (oral feeding followed by removal before digestion), reporting only four minor complications out of 312 events, with no episodes of gastrointestinal reflux or aspiration. Moreover, mothers also reported less stress and high satisfaction with sham feeding [237]. Nonetheless, more research is needed since surgical neonates are at higher risk of feeding intolerance and dysphagia.

Most available data on sham feeding and structured oral interventions derive from studies in preterm or medically ill neonates; evidence specific to surgical infants remains scarce. Therefore, their application in this population should be considered promising but still exploratory.

Indeed, surgery can impair motility [238] and gut microbiome [3, 239]; furthermore, during the postoperative period, neonates often experience negative oral stimulations (like intubation, prolonged EN through oro- or nasogastric tubes, analgesic and sedative medications) [240], and spend little time doing skin-to-skin with their mothers or breastfeeding [3]. Thus, at-risk infants should receive ongoing clinical and instrumental assessments (i.e. video fluoroscopic swallow study, pH-impedance monitoring) [241], to guide tailored interventions and prevent dysphagia.

### Take home messages

1. *The panel suggests that a standardised assessment of oral abilities, potentially using tools like POFRAS, should guide the initiation of oral feeding in neonates with surgical disorders as soon as postoperative intestinal function resumes.*
2. *The panel suggests employing active strategies, such as non-nutritive sucking, oral colostrum, and structured interventions like PIOMI, to promote the development of oral motor skills in surgical neonates, potentially improving feeding outcomes and growth; however, current evidence in this population remains limited, and these approaches should be applied with appropriate clinical caution.*

## Gut microbiota in neonates with gastrointestinal surgical disorders

Recent advances have highlighted the role of gut microbiota in neonatal recovery. In particular, surgical neonates face disrupted microbial colonization due to prematurity, prolonged hospitalization, PN, and antibiotics. These factors delay normal microbiota development [242].

Emerging molecular techniques (including 16S ribosomal RNA gene sequencing and whole-genome metagenomics) allow detailed microbiota profiling in these infants. They often show reduced fewer beneficial anaerobes (e.g. Bifidobacteria and Bacteroides) and more pathogens (e.g. Klebsiella spp., Enterococci, and Staphylococci). Dysbiosis may impair gut barrier, immunity, and metabolism [243–245].

In this context, microbiota profiling can aid diagnosis and treatment. By comparing individual microbial communities to those of age-matched healthy infants, indexes like the microbial dysbiosis index (MDI) quantify deviation from healthy microbiota. Combined with metabolomics, they reveal effects like altered short-chain fatty acids or inflammation. Importantly, microbial signatures may guide risk stratification and targeted interventions (e.g., probiotics, early EN, or microbiota-sparing antibiotics) [246]. In clinical practice, microbiota profiling is beginning to inform targeted interventions in neonatal intensive care; however, current evidence remains limited and largely exploratory. Preliminary data suggest that dysbiosis patterns might help identify infants at higher risk of NEC or feeding intolerance, support tailored probiotic supplementation, and guide the timing and composition of early enteral feeding. Moreover, integrating microbial data into antibiotic stewardship programs may help minimize unnecessary broad-spectrum antibiotic use and preserve microbial diversity, although these approaches still require validation in larger, surgery-specific neonatal cohorts.

The integration of microbiota data into clinical care for surgical neonates represents a promising step toward personalized medicine. Though still evolving, microbiota analysis may soon guide perioperative management. New technologies will help clarify and modulate gut microbiota development [247, 248], even in surgical neonates affected by stress, PN, and early antibiotics [249].

### Take home messages

1. *The panel suggests providing early enteral feeding, if possible with HM, in order to modulate the developing gut microbiota.*
2. *The panel recommends minimizing antibiotic exposure in surgical neonates to preserve gut microbiota balance, reduce dysbiosis, and support optimal immune and intestinal development.*
3. *Microbiota-informed interventions (e.g. targeted probiotics) in surgical neonates, in order to support immune and intestinal development, still require further research.*

## Conclusion

Nutritional care of surgical newborns is complex and needs a multidisciplinary approach before and after surgery. While perioperative fasting is often necessary, this population’s unique metabolic needs and rapid growth necessitate the judicious use of PN to avoid nutritional deficits and the introduction of EN as soon as possible (Table 3).

**Table 3:** Nutritional management by surgical condition: parenteral and enteral nutrition strategies

Condition	Parenteral Nutrition (PN)	Enteral Nutrition (EN)
General post-op care	• Start PN early if EN is not feasible • Avoid hyperglycemia and monitor energy/protein needs; use mixed lipid emulsions	• Initiate EN within 48 h post-op at 10–24 ml/kg/day, progress by 10–30 ml/kg/day based on tolerance. • Prefer mother’s own milk (MOM); use donor human milk (DHM) if MOM unavailable; fortify as needed. • Promote oral feeding
Oesophageal Atresia (OA)	• Total PN required pre-op • Personalize macro- and micronutrient intake by GA and BW	• Start EN 24–48 h post-op via trans-anastomotic tube if placed. In long-gap OA, start early EN via gastrostomy • HM preferred, DHM or formula acceptable.
Congenital Diaphragmatic Hernia (CDH)	• Initiate PN within 24 h; • Aim for ≥120 kcal/kg/day and ≥2.3 g/kg/day protein, balancing fluid restrictions	• Begin EN once bowel function returns. • Use fresh HM as first choice, DHM if unavailable. Consider hydrolysed or amino acid-based formulas in case of reflux or poor tolerance. Fortify or use high-calorie feeds if needed.
Chylothorax	• Total PN for 5–7 days in high-output cases ( > 10 ml/kg/day) • Monitor for protein, fat, immunoglobulin, vitamin losses	• Start EN if drainage < 10 ml/kg/day and patient stable • Use defatted HM or MCT-based formula. Continue fat-modified diet for 2–3 weeks after resolution.
Intestinal Atresia	• Always required pre/post-op; support while awaiting bowel function recovery	• Start EN at 10 ml/kg/day once tolerated. • HM preferred; formula acceptable. In extensive resection (types iiib, IV), use hydrolysed or amino acid-based formulas if needed.
Abdominal Wall Defects (Omphalocele, Gastroschisis)	• Start PN within 24 h of life	• Introduce EN by day 7–12 • Use MOM as first choice, DHM second, formula third. In complex cases or SBS, use hydrolysed or amino acid-based formulas.
Hirschsprung Disease (HD)	• PN needed pre/post-op or when EN is insufficient	• Prefer HM. • In faecal leakage, consider constipating diets; in constipation, soluble fibers like pectin.
Necrotizing Enterocolitis (NEC)	• Phase-dependent PN: 45–55 (acute) to 90–120 kcal/kg/day (recovery)	• Restart EN 5–7 days post-event • Prefer HM (MOM or DHM). Use preterm formula if HM unavailable. In case of intolerance, consider semi-elemental or elemental formulas.
Enterostomy	• Often prolonged PN; monitor sodium, fluids, and growth • Replace losses (albumin, electrolytes) only when clinically indicated.	Start EN within 7 days post-op at < 20 mL/kg/day using HM as first choice; DHM or formula if MOM unavailable. • Adjust EN advancement based on stomal output (SO), diuresis, and peristomal skin integrity: – If SO ≤ 20 mL/kg/day → increase by 10–20 mL/kg/day. – If SO 20–48 mL/kg/day and diuresis > 2 mL/kg/h → increase by ≤ 10 mL/kg/day. – If SO > 48–72 mL/kg/day or skin lesions present → maintain current intake. – If SO > 72 mL/kg/day or diuresis < 2 mL/kg/h → reduce by ~ 10 mL/kg/day. Consider mucous fistula refeeding (MFR) when feasible to enhance intestinal adaptation.
Intestinal Failure (IF)	• Long-term PN with cycling when stable • Prevent IFALD and monitor for complications	• Initiate early EN to promote adaptation. • Use MOM preferentially. If unavailable or not tolerated, use standard formula, hydrolysed, or amino acid-based formulas. Maintain oral stimulation.

Given the limited high-level evidence due to the rarity of these conditions, personalized nutritional strategies, guided by weight trends and hemodynamic stability, are paramount. This position paper, derived from expert opinion across neonatology, paediatric surgery, and paediatric nutrition based on available literature, offer practical tips where strong trial data is lacking.

Future research, particularly well-designed multicentre studies, is essential to establish clearer guidelines for the optimal nutritional support of surgical newborns, ultimately improving their short- and long-term outcomes, including the timely and safe transition to enteral and oral feeding, and preventing complications.

## Data Availability

All data and materials were already published and can be found in the literature.

## Abbreviations

ASPEN: American Society for Parenteral and Enteral Nutrition
BW: Birth weight
CDH: Congenital diaphragmatic hernia
CVC: Central venous catheter
DHM: Donor human milk
ECMO: Extracorporeal Membrane Oxygenation
ELBW: Extremely low birth weight
EN: Enteral nutrition
ERAS: Enhanced Recovery after Surgery
ERNICA: European Reference Network for rare Inherited and Congenital Anomalies
ESPGHAN: European Society of Pediatric Gastroenterology, Hepatology and Nutrition
ESPNIC: European Society of Pediatric and Neonatal Intensive Care
FEF: Full enteral feeding
GA: Gestational age
GR: Gastric residual
HAEC: Hirschsprung-associated enterocolitis
HD: Hirschsprung’s Disease
HE: High enterostomy
HM: Human milk
HMF: Human milk fortification
IFALD: Intestinal associated liver disease
LE: Low enterostomy
LILT: Longitudinal intestinal lengthening and tailoring
MEF: Minimal enteral feeding
MFR: Mucous fistula refeeding
MMA: Methylmalonic acid
MOM: Mother’s own milk
NASPGHAN: North American Society for Pediatric Gastroenterology, Hepatology and Nutrition
NEC: Necrotizing enterocolitis
OA: Oesophageal atresia
OCT: Oral colostrum therapy
PIOMI: Premature Infant Oral Motor Intervention
PN: Parenteral nutrition
PNALD: Parenteral nutrition-associated liver disease
POFRAS: Preterm Oral Feeding Readiness Assessment Scale
PUFAs: Polyunsaturated fatty acids
RCT: Randomised controlled trial
SBS: Short bowel syndrome
SIBO: Small intestine bacterial overgrowth
SICP: Italian Society of Paediatric Surgery
SIN: Italian Society of Neonatology
SINUPE: Italian Society of Paediatric Nutrition
SO: Stomal output
STEP: Serial Transverse Enteroplasty Procedure
TOF: Tracheoesophageal fistula
TRAGI: Transfusion-associated acute gut injury
VLBW: Very low birth weight
